# The application of WHO ICD-PM: Feasibility for the classification of timing and causes of perinatal deaths in a busy birth centre in a low-income country

**DOI:** 10.1371/journal.pone.0245196

**Published:** 2021-01-14

**Authors:** Natasha Housseine, Anne Snieder, Mithle Binsillim, Tarek Meguid, Joyce L. Browne, Marcus J. Rijken

**Affiliations:** 1 Division Woman and Baby, University Medical Centre Utrecht, Utrecht, The Netherlands; 2 Department of Obstetrics and Gynaecology, Mnazi Mmoja Hospital, Zanzibar, Tanzania; 3 Julius Global Health, Julius Centre for Health Sciences and Primary Care, University Medical Centre Utrecht, Utrecht University, Utrecht, The Netherlands; 4 Department of Paediatrics, Mnazi Mmoja Hospital, Zanzibar, Tanzania; 5 Village Health Works, Kigutu, Burundi; University of Mississippi Medical Center, UNITED STATES

## Abstract

**Objective:**

To assess the feasibility of the application of International Classification of Diseases-10—to perinatal mortality (ICD-PM) in a busy low-income referral hospital and determine the timing and causes of perinatal deaths, and associated maternal conditions.

**Design:**

Prospective application of ICD-PM.

**Setting:**

Referral hospital of Mnazi Mmoja Hospital, Zanzibar, United Republic of Tanzania.

**Population:**

Stillbirths and neonatal deaths with a birth weight above 1000 grams born between October 16^th^ 2017 to May 31^st^ 2018.

**Methods:**

Clinical information and an adapted WHO ICD-PM interactive excel-based system were used to capture and classify the deaths according to timing, causes and associated maternal complications. Descriptive analysis was performed.

**Main outcome measures:**

Timing and causes of perinatal mortality and their associated maternal conditions.

**Results:**

There were 661 perinatal deaths of which 248 (37.5%) were neonatal deaths and 413 (62.5%) stillbirths. Of the stillbirths, 128 (31%) occurred antepartum, 129 (31%) intrapartum and for 156 (38%) the timing was unknown. Half (n = 64/128) of the antepartum stillbirths were unexplained. Two-thirds (67%, n = 87/129) of intrapartum stillbirths followed acute intrapartum events, and 30% (39/129) were unexplained. Of the neonatal deaths, 40% died after complications of intrapartum events.

**Conclusion:**

Problems of documentation, lack of perinatal death audits, capacity for investigations, and guidelines for the unambiguous objective assignment of timing and primary causes of death are major threats for accurate determination of timing and specific primary causes of perinatal deaths.

## Introduction

With more than 5 million cases each year, perinatal death remains a significant global health problem. The countries with the highest absolute numbers of stillbirths and neonatal deaths are in Asia and sub-Saharan Africa [1]. Despite increasing attention and investment to address the main causes and to end preventable deaths, perinatal deaths are often poorly recorded and classified in low-income countries [2, 3]. In response to the existence of over 80 widely varying classification systems of perinatal deaths in definition, classification of (underlying) causes of death, comprehensiveness, utilization, accessibility, reliability and alignment to WHO’s International Classification of Diseases (ICD), the ‘WHO application of ICD-10 to perinatal mortality’ (ICD-PM) was developed [4, 5]. ICD-PM is based on the 10th revision of the International Statistical Classification of Diseases and Related Health Problems (ICD-10). Its main purpose is to internationally harmonise the classification of perinatal deaths and produce data that can be used for targeting programmes that address perinatal mortality [5, 6]. The three distinct features of the ICD-PM are 1) the capture of timing of death (antepartum, intrapartum or neonatal), 2) the multilayered approach for classification of the causes of death reflecting varying levels of available information depending on the setting, and 3) linking of the contributing maternal condition to the perinatal death. After pilot studies in middle-income country South Africa (SA) and the United Kingdom (UK), the ICD-PM was identified as a globally applicable perinatal death classification system [6–8]. However, there is very limited experience of the use of ICD-PM in low-income countries, where the burden of perinatal deaths is greatest.

Since the WHO recommends using the ICD-PM on a global scale, we evaluated the feasibility of ICD-PM application in Zanzibar’s tertiary hospital, a busy birth centre in a low-income country setting.

## Methods

### Study design

This study of perinatal deaths was linked to a prospective study of pregnant women who delivered between October 16^th^ 2017 to May 31^st^ 2018 at Mnazi Mmoja Hospital (MMH), Zanzibar, Tanzania.

### Study setting

MMH is the only tertiary care hospital of Zanzibar and provides comprehensive obstetric and neonatal care around the clock. Approximately 11500 women deliver annually. The department was under-resourced and understaffed with an average ratio of birth attendants to labouring women of 1:4 [9]. The stillbirth rate was 39 per 1000 total births and the neonatal mortality was unknown [10]. Intrapartum care was mainly provided in three shared labour rooms and three private delivery rooms. Intermittent auscultation with Pinard, hand-held Doppler and sometimes ultrasound was used for foetal heart rate assessment on admission and throughout labour. Babies with low Apgar scores, birth asphyxia, birthweights < 1500 grams or delivered by caesarean sections (CS) were referred to the upstairs Neonatal Intensive Care Unit (NICU) with a handover sheet. The NICU consists of three rooms: the neonatal care room with four radiant heaters, seven incubators and six cots; the observation room with 14 cots, and the kangaroo care room with eight beds. Two separate perinatal death certificates/notification forms existed. The first form recorded stillbirths and newborns who died immediately after birth in the maternity ward. It provided the following information: the parents’ names, address and occupation, sex of the baby, type of pregnancy (single or multiple), date of birth, birth weight and whether the baby was born dead or alive. The second was a national WHO-adapted death certificate form that captured neonatal deaths in the NICU and recorded the mother’s name and address, date and place of death as well as causes of death. In general, they were filled by nurse-midwives and doctors respectively. Once a week the neonatal deaths were discussed in the perinatal mortality meeting. Stillbirths were usually classified according to appearance of skin changes as either macerated or fresh, as commonly practised in low-income countries [11]. No routine investigations were performed to establish perinatal cause of death.

### Patient selection

Cases of perinatal death were identified using the pre-existing MMH death certificates and selected using predefined inclusion- and exclusion criteria. All stillbirths and neonatal deaths born in MMH who died before discharge from the hospital with a birth weight above 1000 gram were included [1]. Perinatal deaths with a birth weight below 1000 grams were excluded. An exception was made for twins; if one of the babies weighed more than 1000 grams and the other one less, both babies were included. Also, deaths with an unknown birthweight were included in the study because babies weighing less than 1000 grams were not issued a birth notification or death certificate as they were considered miscarriages. Home deliveries, births before arrival to the maternity unit and referred neonates were excluded since the timing, cause of death and maternal condition would be impossible to determine.

### Sources of information

The total number of live births was obtained from the hospital birth notification forms. The stillbirths and neonatal deaths were identified mainly using the MMH death certificates, which were routinely completed by the nurses. Additional stillbirths were manually searched through patient files and the hospital registry book. Determination of the timing and cause of death used information gathered from multiple sources such as maternal and neonatal files, nurses’ reports, attending clinical meetings and perinatal and maternal mortality audits. Socio-demographic information, obstetric history and pregnancy characteristics were obtained from maternal and neonatal files. All perinatal deaths and the cases were anonymized with unique codes.

### Application of the ICD-PM

Two reviewers (AS and NH) independently reviewed the information available and used the ICD-PM three-step to first assign the timing of death as antepartum, intrapartum or postpartum. Subsequently, the cause of death was assigned to one of the six, seven or eleven groups of ICD-PM perinatal cause of death under the antepartum, intrapartum and neonatal groups, respectively [5]. The main ICD-PM perinatal cause of death was then linked to ICD-10 codes of broad and specific causes of death. The third step in the process was to identify the main maternal condition or disease affecting the fetus or infant. Five main maternal groups exist and each group has subgroups to specify the maternal condition. The main perinatal and/or maternal condition was defined as the condition that started the chain of events leading to the death.^1^ The definitions in S1 Table were used to standardise the classification. An obstetrician (MJR) helped resolve any disagreement between the two reviewers.

The WHO recommended interactive based Excel system (S1 Fig), ICD-PM documentation provided by WHO, and ICD-10 were used to extract and classify the perinatal deaths [5]. The excel system contains the minimum set of indicators for perinatal deaths, timing and ICD-PM and ICD-10 codes of causes of death. To assist the classification, we added extra columns in the excel system for the following information: mother’s age, fresh/macerated stillbirth, foetal heart rate on admission (pre- or intrahospital death), cervical dilatation on admission, whether the partograph was available and used, the mother’s haemoglobin (Hb) on admission and the Apgar score after one and five minutes. As the appearance of maceration is not an accurate method of determining timing of stillbirth, various indicators were triangulated in order to classify the timing of stillbirth as antenatal or intrapartum: the presence/absence of foetal heart rate or foetal heart activity on admission and/or during the course of labour, and the physical appearance at birth (i.e. fresh or macerated).

When the timing remained unclear, an attempt was still made to determine the cause of death and the presence of maternal complications. As almost half of the women in Tanzania are anaemic and the prevalence is highest in Zanzibar [12], we classified only severe anaemia (Hb <7.0 g/dl) as a maternal condition of ICD-PM group M4: maternal medical and surgical conditions (maternal circulatory and respiratory diseases) [13].

### Feasibility

To assess the extent to which the ICD-PM classification can be practically carried out in this low-resource tertiary hospital, data sources were identified and challenges during data collection and classification process were systematically recorded [14].

### Analysis

Simple descriptive data analysis consisted of the mean (standard deviation (SD)), median (interquartile range (IQR)), and frequency (percentages) in SPSS version 23.

### Details of ethics approval

This study was approved by the Zanzibar Medical Research Ethics Committee (ZAMREC/0004/AGUST/17). All information was retrieved from hospital records and thus no consent was sought from included women.

## Results

### Baseline characteristics

**Fig 1:**
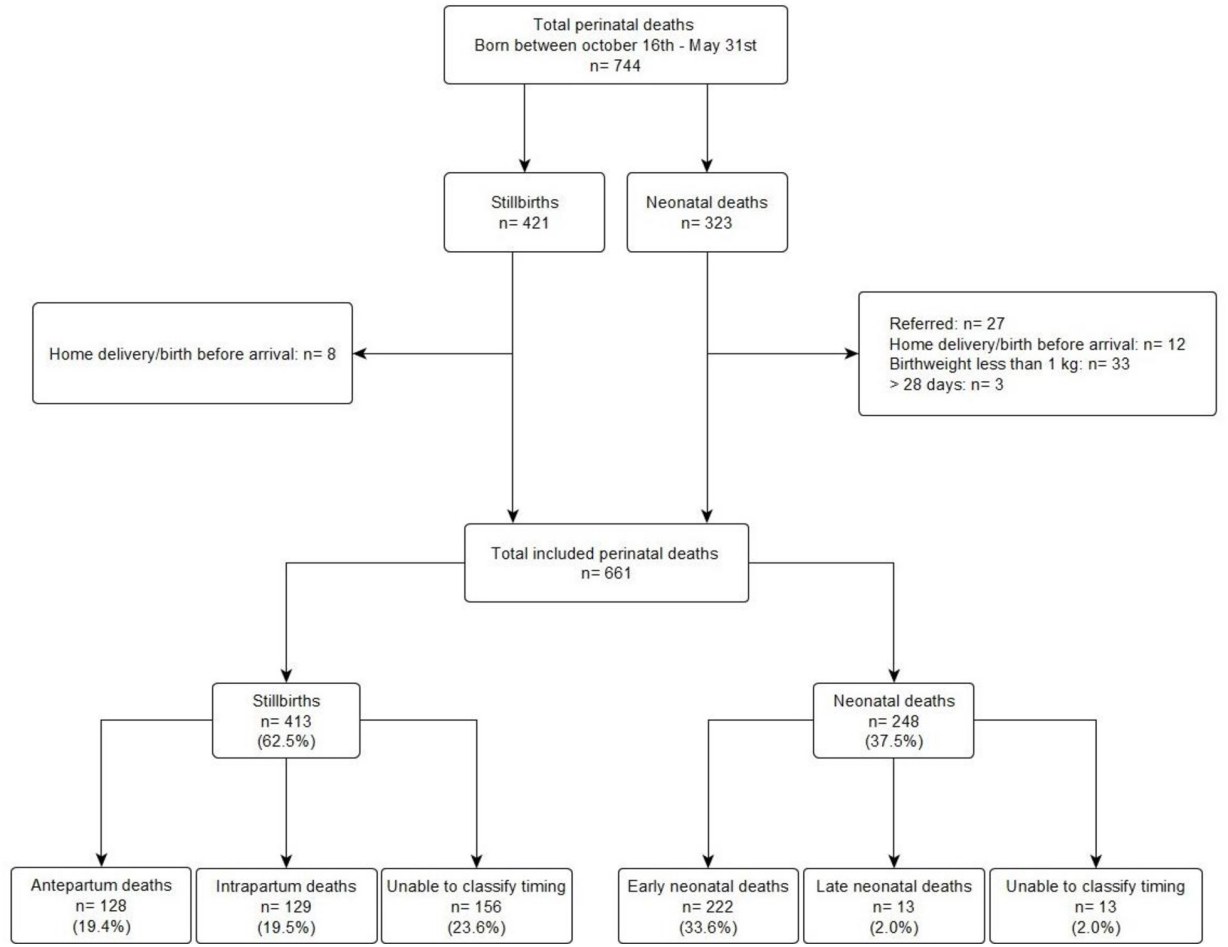
Flowchart of perinatal deaths.

There were 9333 total births retrieved from the birth notification forms between October 16^th^ 2017 to May 31^st^ 2018. Of the 744 perinatal deaths found, 661 had a birthweight of ≥1000g and were born in MMH during the study period (Fig 1). This corresponded to an overall hospital-based perinatal death rate of 71 per 1000 total births (stillbirth rate of 44 per 1000 total births, n = 413 and neonatal death rate of 27 per 1000 live births, n = 248).

Baseline characteristics of the perinatal deaths are shown in Table 1. The mean age of the mothers was 28.8 (SD 6.5) years and the median parity one (IQR 0–3). The median number of ANC visits was 2 (IQR 1–5). The overall maternal HIV status was 0.9% (n = 6/661). The mean birthweight of perinatal deaths was 2434 (SD 958) grams.

**Table 1:** Baseline characteristics of perinatal deaths.

*Variable*		Stillbirths n = 413 (62.5)	Neonatal deaths n = 248 (37.5)	All n = 661 (100)
*Age*	Mean (SD)	29.1 (6.6)	28.0 (6.2)	28.8 (6.5)
*Residence*	Urban	103 (24.9)	76 (30.6)	179 (27.1)
	Mixed	149 (36.1)	79 (31.9)	228 (34.5)
	Rural	80 (19.4)	49 (19.8)	129 (19.5)
	Unknown	81 (19.6)	44 (17.7)	125 (18.9)
*Gravidity*	Median (IQR)	3 (1–5)	2 (1–4)	2 (1–5)
*Parity*	Median (IQR)	2 (0–4)	1 (0–3)	1 (0–3)
*Number of ANC visits*	0	0 (0.0)	0 (0.0)	0 (0.0)
	1–3	158 (38.3)	50 (20.2)	208 (31.5)
	4 or more	104 (25.2)	54 (21.8)	158 (23.9)
	Unknown	151 (36.6)	143 (57.6)	294 (44.5)
*Type of pregnancy*	Singleton	376 (91.0)	179 (72.2)	555 (84.0)
	Twin	25 (6.1)	35 (14.1)	60 (9.1)
	Higher order multiple	2 (0.5)	3 (1.2)	5 (0.8)
	Unknown	10 (2.4)	31 (12.5)	41 (6.2)
*Maternal HIV status*	Positive	4 (1.0)	2 (0.8)	6 (0.9)
	Negative	271 (65.6)	173 (69.8)	444 (67.2)
	Unknown	138 (33.4)	73 (29.4)	211 (32.0)
*Gestational Age*	Preterm	98 (23.7)	78 (31.5)	176 (26.6)
	Term	47 (11.4)	49 (19.8)	96 (14.5)
	Postterm	12 (2.9)	4 (1.6)	16 (2.4)
	Unknown/fundal height only	256 (62.0)	117 (47.2)	373 (56.4)
*Method of GA assessment*	LMP	77 (18.6)	43 (17.3)	120 (18.2)
	USS	73 (17.7)	50 (20.2)	122 (18.5)
	Clinical examination/FH	129 (31.2)	60 (24.2)	189 (28.6)
	Unknown	134 (32.4)	95 (38.3)	229 (34.6)
*Mode of delivery*	SVD	243 (58.8)	137 (55.2)	380 (57.5)
	Vacuum	5 (1.2)	4 (1.6)	9 (1.4)
	Caesarean section	63 (15.3)	73 (29.4)	136 (20.6)
	Unknown	102 (24.7)	34 (13.7)	136 (20.6)
*Sex*	Male	216 (52.3)	136 (54.8)	352 (53.3)
	Female	187 (45.3)	110 (44.4)	297 (44.9)
	Unknown	10 (2.4)	2 (0.8)	12 (1.8)
*Birthweight (g)*	Mean (SD)	2439 (924)	2425 (1013)	2434 (958)
	<2500	180 (43.6)	112 (45.2)	292 (44.2)
	2500–3999	153 (37.0)	85 (34.3)	238 (36.0)
	≥4000	22 (5.3)	18 (7.3)	40 (6.1)
	Unknown	58 (14.0)	33 (13.3)	91 (13.8)

Values presented as number(percentage) unless otherwise specified.

ANC = antenatal care, GA = gestational age, LMP = last menstrual period, USS = ultrasonography, FH = fundal height, SVD = spontaneous vaginal delivery.

### Timing of perinatal deaths

Of all 661 perinatal deaths, 62.5% (n = 413) were stillbirths of which 31.0% (n = 128) were antepartum, 31.2% (n = 129) intrapartum and 37.8% (n = 156) of unknown timing; and 37.5% (n = 248) were neonatal deaths that occurred before hospital discharge. Of the 156 stillbirths of ‘unable to classify the timing’, nearly two-thirds (63.5%, n = 99) were due to missing maternal files (Fig 1 and Table 2).

**Table 2:** ICD-PM main groups of perinatal causes of deaths and associated maternal conditions.

Maternal condition	M1: Complications of placenta, cord and membranes	M2: Maternal complications of pregnancy	M3: Other complications of labour and delivery	M4: Maternal medical and surgical conditions	M5: No maternal condition identified	Unable to classify	Total (%)
**Perinatal cause of death**							
			**Antepartum death (A)**				
A1: Congenital malformations, deformations and chromosomal abnormalities	0	1	0	1	2	0	4 (3.1)
A2: Infection	0	0	0	1	0	0	1 (0.8)
A3: Antepartum hypoxia	19	1	1	38	0	0	59 (46.1)
A4: Other specified antepartum disorder	0	0	0	0	0	0	0 (0.0)
A5: Disorders related to foetal growth	0	0	0	0	0	0	0 (0.0)
A6: Foetal death of unspecified cause	5	9	0	2	48	0	64 (50.0)
**Total (%)**	24 (18.8)	11 (8.6)	1 (0.8)	42 (32.8)	50 (39.1)	0 (0.0)	**128 (100)**
			**Intrapartum death (I)**				
I1: Congenital malformations, deformations and chromosomal abnormalities	0	1	0	0	1	0	2 (1.6)
I2: Birth trauma	0	0	0	0	0	0	0 (0.0)
I3: Acute intrapartum event	27	2	24	26	8	0	87 (67.4)
I4: Infection	0	0	0	0	0	0	0 (0.0)
I5: Other specified intrapartum disorder	0	0	0	0	0	0	0 (0.0)
I6: Disorders related to foetal growth	0	1	0	0	0	0	1 (0.8)
I7: Intrapartum death of unspecified cause	0	5	3	0	31	0	39 (30.2)
**Total (%)**	27 (20.9)	9 (7.0)	27 (20.9)	26 (20.2)	40 (31.0)	0 (0.0)	**129 (100)**
			**Stillbirths of unknown timing**				
**Unable to classify (%)**	7 (4.5)	2 (1.3)	2 (1.3)	11 (7.1)	35 (22.4)	99 (63.5)	**156 (100)**
			**Neonatal death (N)**				
N1: Congenital malformations, deformations and chromosomal abnormalities	1	4	0	2	12	0	19 (7.7)
N2: Disorders related to foetal growth	0	0	0	0	1	0	1 (0.4)
N3: Birth trauma	0	0	0	0	0	0	0 (0.0)
N4: Complications of intrapartum events	17	2	25	16	38	0	98 (39.5)
N5: Convulsions and disorders of cerebral status	0	0	0	0	0	0	0 (0.0)
N6: Infection	1	0	1	4	7	0	13 (5.2)
N7: Resp. and cardiovascular disorders	3	4	1	9	17	1	35 (14.1)
N8: Other neonatal conditions	0	0	0	1	1	1	3 (1.2)
N9: Low birth weight and prematurity	2	10	0	5	5	0	22 (8.9)
N10: Miscellaneous	0	0	0	0	1	0	1 (0.4)
N11: Neonatal death of unspecified cause	0	1	1	1	9	0	12 (4.8)
Unable to classify	0	0	0	1	5	38	44 (17.7)
**Total (%)**	24 (9.6)	21 (8.5)	28 (11.3)	39 (15.7)	96 (38.7)	40 (16.1)	**248 (100)**
**Total perinatal deaths (%)**	82(12.4)	41(6.2)	58(8.8)	118(17.9)	223(33.7)	139(21.0)	661(100)

### Causes of perinatal deaths and associated maternal conditions

The most common cause of perinatal death was hypoxia (Table 2). Hypoxia was classified as antepartum hypoxia for the antepartum deaths (46.1%, n = 59/128), as acute intrapartum events for the intrapartum deaths (67.0%, n = 87/129) and as complications of intrapartum events for the neonatal deaths (39.5%, n = 98/248).

A third of mothers had no associated maternal complications (33.7%, n = 223/661). The most common conditions were maternal medical and surgical conditions (17.9%, n = 118/661), mainly hypertensive disorders (88.2%, n = 105/118) and complications of placenta, cord and membranes (12.4%, n = 82/661). There were sixteen maternal deaths associated with perinatal deaths. Files were missing in 21.0% (n = 139/661) of mothers so maternal conditions could not be assigned for these cases.

### Antepartum

Of the 128 antepartum deaths, half (50.0%, n = 64/128) were classified as foetal deaths of unspecified cause, mostly to mothers without an identified condition (78.1%, n = 50/64). In 46.1% (n = 59/128) death followed after antepartum hypoxia. There were two main associated maternal conditions in these cases: maternal medical and surgical complications (64.4%, n = 38/59), mainly hypertensive disorders, and complications of placenta, cord and membranes such as placental abruption and praevia (32.2%, n = 19/59) (Table 2 and S1 Text: Case 5).

### Intrapartum

Of the 129 deaths that occurred intrapartum most followed an acute intrapartum event (67.4%, n = 87/129). These deaths were often associated with a maternal condition (90.1%, n = 79/87): complications of placenta, cord and membranes (31.0%, n = 27/87) mainly placental abruption and cord prolapse; maternal medical and surgical conditions (29.9%, n = 26/87), often hypertensive disorders; and other complications of labour and delivery such as malpresentation, malposition and disproportion and uterine rupture (27.6%, n = 24/87). In addition, 30.2% (n = 39/129) deaths were of unspecified cause often with unknown events between the last foetal heart rate and delivery (Table 3 and S1 Text: Case 6). No maternal complication was identified in 79.5% (n = 31/39) of the mothers with stillbirths of unspecified cause and in 31.0% (n = 40/129) of all intrapartum deaths (Table 2).

**Table 3:** Issues in implementing ICD-PM in a low-resource setting.

Issue	Description	Potential solutions
Unable to classify timing of death due to missing or conflicting data	Poor monitoring or documentation of: foetal heart rate (FHR), maceration, and cervical dilation e.g.:	At a clinical level: FHR (on admission and labour) and stillborn appearance should be assessed and documented accurately. Filing system in place for proper storage of files.
	• Inadequate intrapartum assessment of women who arrive in early or advanced stage of labour (S1 Text: Cases 2 and 3).	ICD-PM classification system: Develop standardised and operationalised definition for determining timing of death as antepartum and intrapartum stillbirths.
	• No FHR or appearance of stillbirth recorded, only Apgar score	
	• Conflicting data of FHR and maceration stillborn baby (fresh/macerated).	
	• Only one FHR usually recorded in multiple gestation (S1 Text: Case 4).	
	• Vaginal examination not performed, mostly due to per vaginal bleeding.	
	• Missing files	
Unable to assign code to perinatal deaths of unknown timing	Determining timing of death is a pre-requite to assigning ICD-PM categories of perinatal causes of death; if timing is unknown you cannot assign cause of death. (S1 Text: Cases 1–4)	Develop a new category for perinatal death of unknown timing e.g. as suggested by Aminu et al.^14^
High proportion of antepartum deaths of Unspecified causes	A high proportion of antepartum deaths were of unspecified causes and no identified maternal condition (S1 Text: Case 5). This may be due to missing data. However, similar findings were seen across settings (including high income countries).	ICD-PM classification system: difficult to make suggestions since other perinatal death classifications also have high proportion of unspecified antepartum death.
		At a clinical level: improve history taking, physical examination and investigations.
		ICD-PM classification system: especially for low resource setting it may be programmatically useful to identify modifiable causes of death in the antenatal period
Intrapartum deaths of Unspecified cause of death	A high proportion of intrapartum deaths were of unspecified causes which could be related to suboptimal quality of intrapartum care (S1 Text: Case 6).	Modifiable causes are not captured in ICD-PM system. Especially for low resource setting it may be programmatically useful to include a separate category for modifiable causes such as delay in monitoring and intervening.
Variable interpretation of causes of death	This occurs when there are competing perinatal conditions present e.g. in cases of birth asphyxia and meconium aspiration; prematurity/low birth and respiratory and circulatory disorders (S1 Text: Case 7).	Although ICD-10 defines main cause of death as the condition that started the chain of events leading to the death, it can be difficult to determine primary cause. Thus, further guidance and criteria for assigning cause of perinatal death may be required as suggested by Goldenberg et al.^23^
	There may also be competing maternal conditions present e.g. the presence of hypertension and placental abruption /twin pregnancy/complications of labour and delivery (S1 Text: Cases 8 and 9)	Training of staff to use ICD-PM may reduce subjectivity.
Unable to classify ICD-10 specific categories of causes of death	After identifying ICD-PM broad groups of causes of perinatal death, it is important to identify specific causes of death but missing data limited further classification.	At a clinical level: improved history taking, physical examination, documentation and investigations.

### Neonatal deaths

The neonatal deaths were classified as either early (89.5%, n = 222/248) or late neonatal deaths (5.2%, n = 13/248). The most common perinatal causes of death were complications of intrapartum events, including birth asphyxia and intrauterine hypoxia (39.5%, n = 98/248) and respiratory and cardiovascular disorders (14.1%, n = 35/248). No maternal complication was identified for 38.7% (n = 96/248) of mothers; 15.7% (n = 39/248) had a maternal medical and surgical condition, commonly hypertensive disorders (Table 2).

### Feasibility of ICD-PM implementation

**Fig 2:**
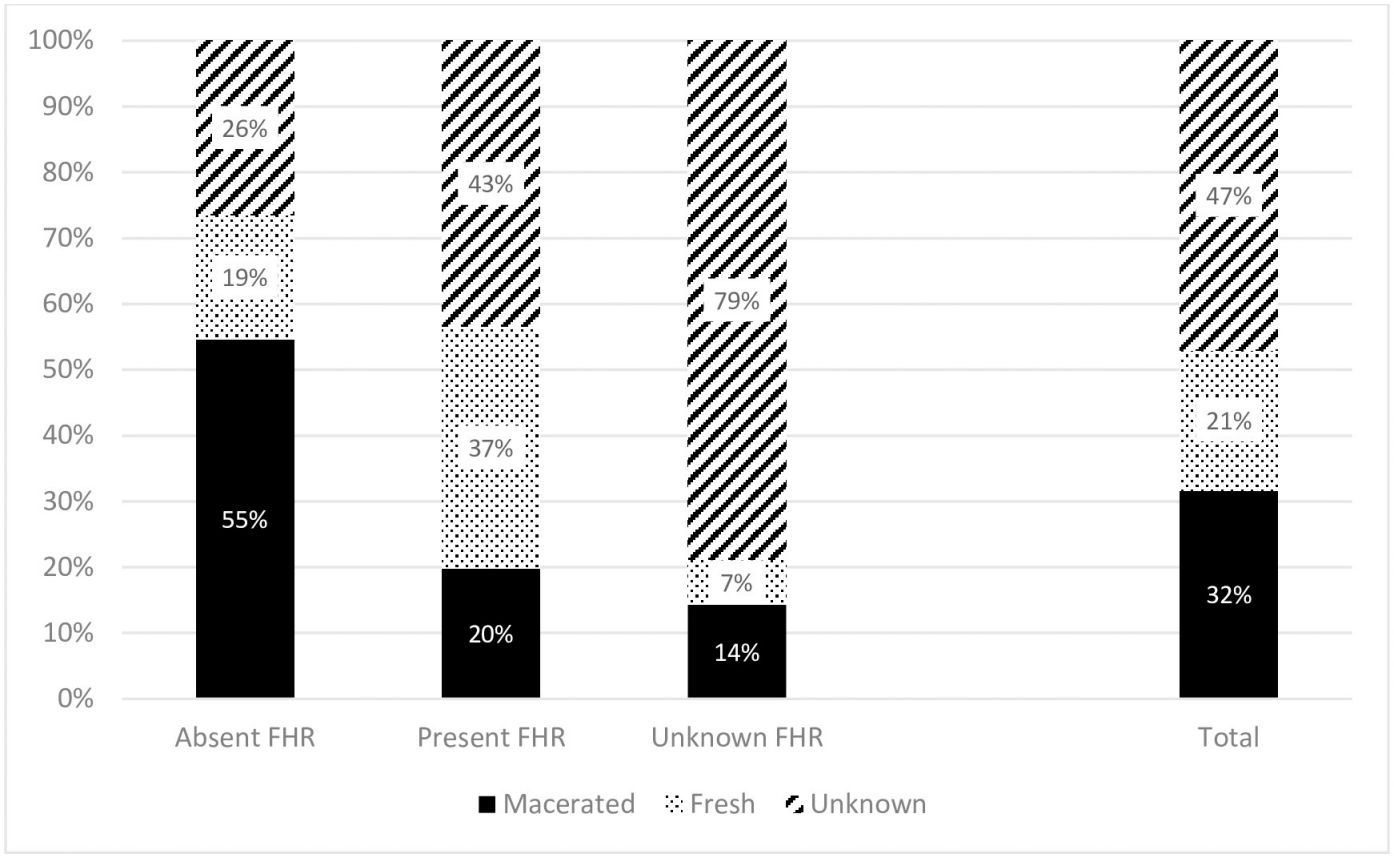
Proportions of macerated and fresh stillbirths and foetal heart rate or foetal heart activity detection on admission.

Issues with implementing ICD-PM in this setting are highlighted in Fig 2, Table 3 and are also illustrated with specific examples in S1 Text. The most common reason for ‘unable to classify’ either the timing or cause was missing files. Also, there were difficulties in determining the timing of perinatal death especially due to non-documentation and conflicting evidence. For example, 22% (n = 93) of stillbirths had no documentation of both foetal heart rate and maceration. Also, the appearance of stillbirth (macerated/fresh) was unknown in nearly half of the number of cases (47%, n = 194) and thus the recording of foetal heart rate classified more stillbirths. In addition, 14% (57) of stillbirths had conflicting evidence of foetal heart rate and skin appearance (macerated/fresh, Fig 2 and S1 Text: Case 1).

Assigning the primary cause of death in neonates with multiple events was challenging. For example, in babies with very low Apgar score after birth and meconium-staining of the liquor, we consistently assigned birth asphyxia over meconium aspiration as the cause of death because it was difficult to differentiate between the two (S1 Text: Case 7). It was also difficult to diagnose intrauterine growth restriction because gestational age was often unknown (56.4%, n = 373/661) due to unknown last menstrual period and absent (early) dating scan. In addition, problems were encountered in assigning specific ICD-PM maternal conditions considered to contribute most to the perinatal death when competing conditions were present e.g. abruption placenta was often associated with hypertensive disorders (Table 3 and S1 Text: Cases 8 and 9).

## Discussion

### Main findings

Using a cohort of 661 perinatal deaths, we evaluated the feasibility of ICD-PM application in a busy birth centre in a low-income country setting. Nearly two-fifths were neonatal deaths, and three-fifths were stillbirths. A large proportion of stillbirth (38%) had unknown timing and of those with known timing, half occurred antepartum and half were intrapartum. Half of the number of antepartum deaths were unexplained and 67.4% and 39.5% of intrapartum and neonatal deaths, respectively, occurred after intrapartum events. Often mothers were healthy across all three time periods, although the most frequent maternal complications were medical and surgical conditions (mainly hypertensive disorders (16%)) and complications of placenta cord and membranes. Although useful in capturing main causes of death and associated maternal conditions, the feasibility of using ICD-PM and other perinatal death classification systems in a busy low-resource hospital, like MMH, is hugely dependent on diagnostic capacity, documentation and record-keeping practices.

### Strengths and limitations

We applied the ICD-PM to all perinatal deaths in a busy referral birth centre in a low-income country. In doing so, we carried out the largest ICD-PM study to date that includes both stillbirths and neonatal deaths in a low-resource setting. We also clearly demonstrated in detail, with specific examples, the issues that exist in implementing the ICD-PM classification and offered recommendations at various levels to improve its applicability in in low-resource settings (Fig 2, Table 3 and S1 Text). We were able to accurately determine the perinatal mortality rate in this hospital and assign main causes of death. Prior to this study the neonatal mortality rate was not available. The independent review process reduced misclassification. However, there was a much higher rate of missing information compared to other ICD-PM studies. There was no filing system in place leading to misplacement of files and as such the most common reason for ‘unable to classify’ was missing maternal files. Missing information was also due to inadequate assessment and undocumented observation, e.g. of foetal heart rate, low Apgar score, birth weight and maceration/fresh [15]—which is also a likely contributor to poor birth outcomes. Also, in this hospital, due to the high number of stillbirths and neonatal deaths, only selected neonatal deaths were discussed in audit meetings which were also held and attended irregularly and many times lacked the people involved in routine care [16]. The issue of missing information likely caused misclassification of timing and causes of perinatal deaths. Efforts were made to reduce the rate of missing data by using multiple data sources including: maternal files, death certificates, clinical and audit meetings and daily report books. In addition, the results obtained and the need to improve clinical documentations and record-keeping were shared with hospital staff during perinatal deaths audits.

### Interpretation

Tanzania is a major contributor to the global burden of perinatal and maternal mortality [17]. The institutional perinatal death rate of this referral hospital was very high, and therefore the rates highlighted in this manuscript were not comparable to national levels [18]. While the stillbirth rate was comparable to those found in various Sub-Saharan Africa hospitals, the neonatal death rate was much higher in this high-volume low-resource referral hospital [19]. An overview of timing and causes of perinatal deaths in ICD-PM studies performed in low-, middle- and high-income countries is shown in S2 Table [6, 20–22]. In these studies, in the UK and SA where women received better intrapartum care, almost all stillbirths were antepartum (91%, 81–82%), and in line with our findings, at least half of stillbirths in low-income countries were intrapartum (51–78%)–a picture that resembles national and global estimates [17, 23–25]. Although the rates vary, stillbirth classifications, including ICD-PM, show that most antepartum deaths were of unspecified cause and without identified maternal condition particularly in low and middle income countries (50–89%) [3, 17, 26]. This presents a global challenge in identifying causes of perinatal deaths and targeted interventions in the antenatal period.

In our study, the perinatal cause of death was often hypoxia which is not useful in identifying and addressing causes of death. However, we identified hypertensive disorders and ‘complications of placenta, cord and membranes’ as important maternal conditions associated with perinatal deaths across all periods—these findings are similar to other studies including ICD-PM [17]. Thus, linking perinatal deaths to maternal conditions in the ICD-PM classification identifies areas for interventions to prevent perinatal deaths—a major strength of ICD-PM [3, 6, 17, 26]. Similar to other studies in low resource setting s, disorders related to fetal growth (A3, I6 and N9) were hard to assign to a specific group, as the lack of early ultrasound for accurate dating of pregnancy makes it difficult to detect growth restriction [22]. However, prematurity and/or low birth weight was an important cause of neonatal death across all the studies (29–37%) which emphasizes the fact that preterm labour is a global target for intervention to reduce perinatal deaths [27, 28]. Specifically, for low and middle-income countries, the high numbers of stillbirths and neonatal deaths related to intrapartum causes identifies this period as highest risk and for quality improvement programmes [3, 15, 29, 30].

Subjectivity of methods used for classification across studies and varying quality of data may partly explain the differences observed between ICD-PM studies. While ICD-PM is readily applicable to settings with established country-wide perinatal surveillance and classification systems, challenges exist in accurate application in low-income settings [6, 20–22]. In low-income countries, missing information on obstetric history and clinical data presents a widespread threat to the application of the various perinatal classifications and identifying specific causes of death with up to 57.4% of stillbirths remaining unclassified [21, 22, 26]. Missing files, undocumented or conflicting evidence between foetal heart rate and appearance of stillborn (macerated/fresh) leads to problems in the determination of the timing of stillbirths (Table 3)–the first step in the ICD-PM classification without which assigning ICD-PM perinatal cause of death is impossible [11, 15, 22, 31]. Studies showed that foetal heart assessment by auscultation, particularly on admission, was a reliable means of determining foetal viability and timing of stillbirth [15, 16, 32]. Yet, as we have also shown in previous studies, conflict of foetal heart rate detection and skin appearance of stillbirth occur and is preventable with adequate assessment e.g. of foetal heart rate on admission [9, 15].

Although death certificates and perinatal death audits are central in gathering information for ICD-PM classification [5, 33], many neonatal deaths and stillbirths are still not recorded or issued death certificates in low resource settings [33]. This is not the case in MMH hospital where we commend the use of death certificates and distinction between stillbirths and neonatal deaths. However, death certificates were often incomplete, and missing relevant perinatal indicators such as gestational age, birth weight, causes of stillbirths and maternal complications. Apart from obstetric history and clinical data, accurate determination of the cause of death may also require laboratory tests, imaging, and autopsy which were (commonly) absent in all ICD-PM studies in low-income countries [34], making it more difficult if not impossible to accurately determine important and more specific causes of death such as infections and congenital anomalies [34–36]. A study in Mozambique found that minimally invasive tissue sampling showed a large concordance with complete autopsy and could be useful for the determination of the cause of death in low-income countries [37].

Other challenges may also be inherent to the ICD-PM and other classification systems. There was ambiguity in assigning primary cause of death when two or more competing primary causes of death or associated maternal complications are applicable e.g. abruption placenta was often associated with hypertensive disorders and birth asphyxia with meconium [34]. Also, an extension of the ICD-PM system is required to capture potentially modifiable factors such as delays in monitoring and providing emergency obstetric and newborn care in these type of settings. It remains to be seen how ICD-PM can be incorporated in perinatal death audits for better capture and analysis of perinatal deaths and continuous quality improvement. With the overwhelming number of deliveries, the lack of staff in this setting remains a major contributing factor to perinatal deaths and is multi-level obstacle limiting the application of perinatal death classifications including ICD-PM in routine clinical practice.

## Conclusion and recommendations

It was possible to determine maternal medical and surgical conditions, hypertensive disorders in particular, and intrapartum events as major causes of both intrapartum stillbirths and neonatal deaths in a busy maternity unit in a low resource setting. However, a high number of perinatal deaths were classified as deaths of unspecified timing and cause. In low-income countries, missing clinical information and investigations are the major threats to perinatal death classifications. Thus, better clinical assessment and documentation from the time of admission, including foetal heart rate, is crucial. There is a need to train additional staff, strengthen death certificate record-keeping and perinatal death audits of both stillbirths and neonatal deaths according to established guidelines, accompanied by the prospective use of the ICD-PM system. Lastly, global applicability of ICD-PM requires standardized operationalized definitions and harmonised guidance on assigning timing of death and primary and contributory causes of death, including when the time of death remains unknown.

## Supporting information

S1 Table Definition of terms used during classification. (DOCX)

S2 Table Main causes of perinatal deaths in the ICD-PM studies. (DOCX)

S1 Fig Modified ICD-PM interactive system used for data extraction and classification. (DOCX)

S1 Text Examples of cases that illustrate challenges in applying ICD-PM classification. (DOCX)
