# Supplementary material for: The application of WHO ICD-PM: Feasibility for the classification of timing and causes of perinatal deaths in a busy birth centre in a low-income country
Source: PLoS One. 2021 Jan 14;16(1):e0245196. doi: 10.1371/journal.pone.0245196 (PMC7808596; doi:10.1371/journal.pone.0245196)
Supplement: S1 Table — (DOCX) [file pone.0245196.s001.docx]

| S1 Table: Definitions of terms used during classification | |
| --- | --- |
| **Term** | **Definition** |
| **Timing of perinatal death** |  |
| **Antepartum** | Foetal death at ≥28weeks of gestation or birth weight ≥1000g that occurred before the start of labour |
| **Intrapartum** | Foetal death at ≥28weeks of gestation or birth weight ≥100g that occurred during childbirth |
| **Neonatal death** | Newborn death that occurred before hospital discharge |
| **Early neonatal death** | Neonatal death that occurred within 7 days of birth |
| **Late neonatal death** | Neonatal death that occurred between 8-28 of birth |
| **Unable to classify the timing** | If no information was available to determine timing, for example, because of missing files, unmeasured foetal heart rate or contradicting evidence |
| **Macerated stillbirth** | A stillbirth that shows skin and soft tissue changes such as redness, peeling, skin discolouration |
| **Fresh stillbirth** | Stillbirth with intact skin and other no signs of maceration |
| **Main perinatal causes of death** | ICD-PM groups of the main condition in the foetus/newborn according to the timing of death |
| **Perinatal death of unspecified cause** | No information is available that would permit a more specific cause of death to be assigned |
| **Unable to classify cause of perinatal death** | Perinatal deaths with missing files and no information for classification |
| **Miscellaneous causes** | Neonatal deaths that appeared to have multiple contributing factors leading to the death and unable to establish the main cause of death |
| **Maternal condition** | ICD-PM groups of main maternal complication that is closely linked as a cause of the perinatal death |
