# Supplementary material for: The application of WHO ICD-PM: Feasibility for the classification of timing and causes of perinatal deaths in a busy birth centre in a low-income country
Source: PLoS One. 2021 Jan 14;16(1):e0245196. doi: 10.1371/journal.pone.0245196 (PMC7808596; doi:10.1371/journal.pone.0245196)
Supplement: S2 Table — (DOCX) [file pone.0245196.s002.docx]

| S2 Table. Main causes of perinatal deaths in the ICD-PM studies (6,20–22) | | | | | | | |
| --- | --- | --- | --- | --- | --- | --- | --- |
|  | | High-income countries | Middle- and low- income countries | | | | |
|  |  | United Kingdom (2016) | South-Africa (2016) | South Africa (2018) | Multi-country* (2019) | Zambia (2019) | Tanzania (Zanzibar, 2020) |
| Number of total deaths | | 9067 perinatal deaths | 689 perinatal deaths | 26810 perinatal deaths | 1267 stillbirths | 75 perinatal deaths | 661 perinatal deaths |
| Timing of death | Antepartum | 48.3% | 50% | 58.2% | 42% | 9.3% | 19.4% |
|  | Intrapartum | 5% | 11% | 14.0% | 50.7% | 33.3% | 19.5% |
|  | Neonatal | 46.7% | 39% | 27.8% | - | 57.3% | 37.5% |
|  | Unable to classify | 0% | 0% | 0% | 7.3% | 0% | 23.6% |
| Main causes antepartum deaths | | Foetal death of unspecified cause (A6) 60%  Congenital malformations, deformations and chromosomal abnormalities (A1) 22% | Antepartum Hypoxia (A3) 53%  Foetal death of unspecified cause (A6) 42% | Foetal death of unspecified cause (A6) 67.5%  Other specified antepartum disorder (A4) 18.9% | Foetal death of unspecified cause (A6) 89%  Infections (A2) 8.6% | Foetal death of unspecified cause (A6) 71%  Antepartum Hypoxia (A3) 14%  Congenital malformations, deformations and chromosomal abnormalities (A1) 14% | Foetal death of unspecified cause (A6) 50.0%  Antepartum Hypoxia (A3) 46% |
| Main causes intrapartum deaths | | Acute intrapartum event (I3) 65%  Intrapartum death of unspecified cause (I7) 26% | Acute intrapartum event (I3) 93% | Acute intrapartum event (I3) 69.2%  Intrapartum death of unspecified cause (I7) 10.0% | Intrapartum death of unspecified cause (I7) 61%  Acute intrapartum event (I3) 31% | Acute intrapartum event (I3) 84%  Congenital malformations, deformations and chromosomal abnormalities (A1) 16% | Acute intrapartum event (I3) 67%  Intrapartum death of unspecified cause (I7) 30% |
| Main causes neonatal deaths | | Low birth weight and prematurity (N9) 32%,  Congenital malformations, deformations and chromosomal abnormalities (N1) 27%  Neonatal death of unspecified cause (N11) 27% | Respiratory and cardiovascular disorders (N7) 35%  Low birth weight and prematurity (N9) 29% | Complications of intrapartum events (N4) 29.3%  Low birth weight and prematurity (N9) 28.5%  Respiratory and cardiovascular disorders (N7) 19.2% |  | Complications of intrapartum events (N4) 44%  Low birth weight and prematurity (N9) 37% | Complication of intrapartum events (N4) 40%  Respiratory and cardiovascular disorders (N7) 14.4% |
| Main maternal condition | | M5: 49.8%  M1:21%  M2: 10.6% | M5: 36%  M4: 25.7%  M3: 17.9% | M4: 32.7%  M5: 31.1%  M1: 17.8% | M1: 26.7%  M3: 25.5%  M5: 23.7% | M3: 53.5%  M5: 34.9%  M2: 9.3% | M5: 33.7%  M4: 17.9%  M1: 12.4% |
| *Kenya, Malawi, Sierra Leone and Zimbabwe  **Abbreviations:** M1= Complications of placenta, cord and membranes**,** M2= Maternal complications of pregnancy**,** M3=Other complications of labour and delivery, M4= Maternal medical and surgical conditions, M5= No maternal condition identified  **Search string in PubMed on 30/01/2020:**  ((("stillbirth"[MeSH Terms] OR "stillbirth"[All Fields]) OR ("perinatal death"[MeSH Terms] OR ("perinatal"[All Fields] AND "death"[All Fields]) OR "perinatal death"[All Fields] OR ("perinatal"[All Fields] AND "deaths"[All Fields]) OR "perinatal deaths"[All Fields])) OR ("perinatal death"[MeSH Terms] OR ("perinatal"[All Fields] AND "death"[All Fields]) OR "perinatal death"[All Fields] OR ("neonatal"[All Fields] AND "deaths"[All Fields]) OR "neonatal deaths"[All Fields])) AND ((("classification"[Subheading] OR "classification"[All Fields] OR "classification"[MeSH Terms]) OR ("international classification of diseases"[MeSH Terms] OR ("international"[All Fields] AND "classification"[All Fields] AND "diseases"[All Fields]) OR "international classification of diseases"[All Fields])) OR ICD-PM[All Fields]) AND (("2016/01/01"[PDAT] : "2020/01/31"[PDAT]) AND "humans"[MeSH Terms]) | | | | | | | |
