# Supplementary figures and images for: The application of WHO ICD-PM: Feasibility for the classification of timing and causes of perinatal deaths in a busy birth centre in a low-income country
Source: PLoS One. 2021 Jan 14;16(1):e0245196. doi: 10.1371/journal.pone.0245196 (PMC7808596; doi:10.1371/journal.pone.0245196)

S1
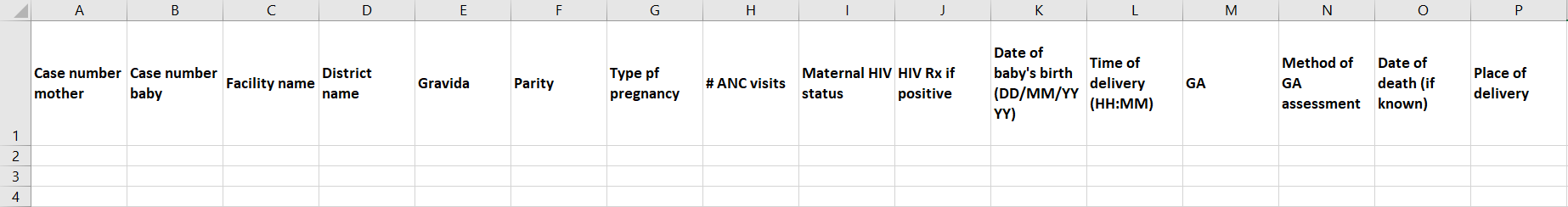
 Fig. Modified ICD-PM interactive system used for data extraction and classification


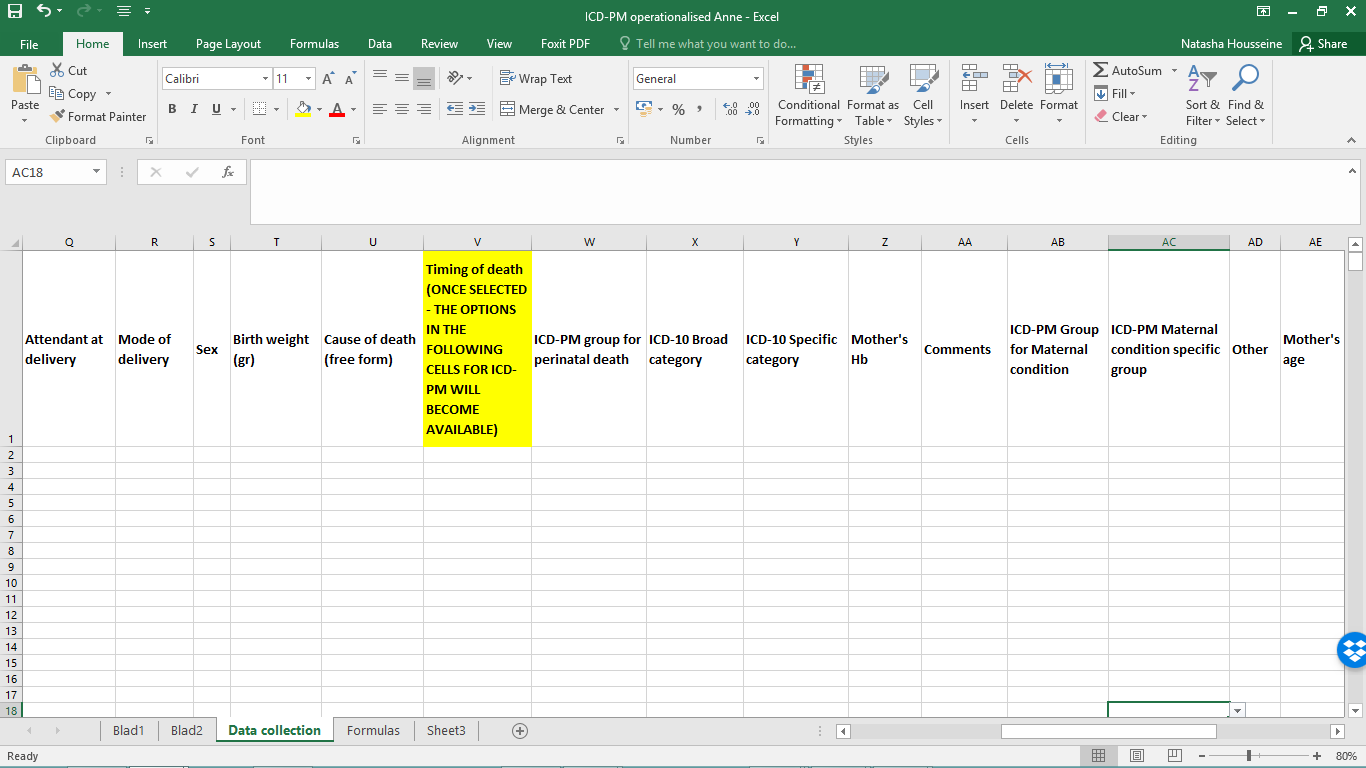


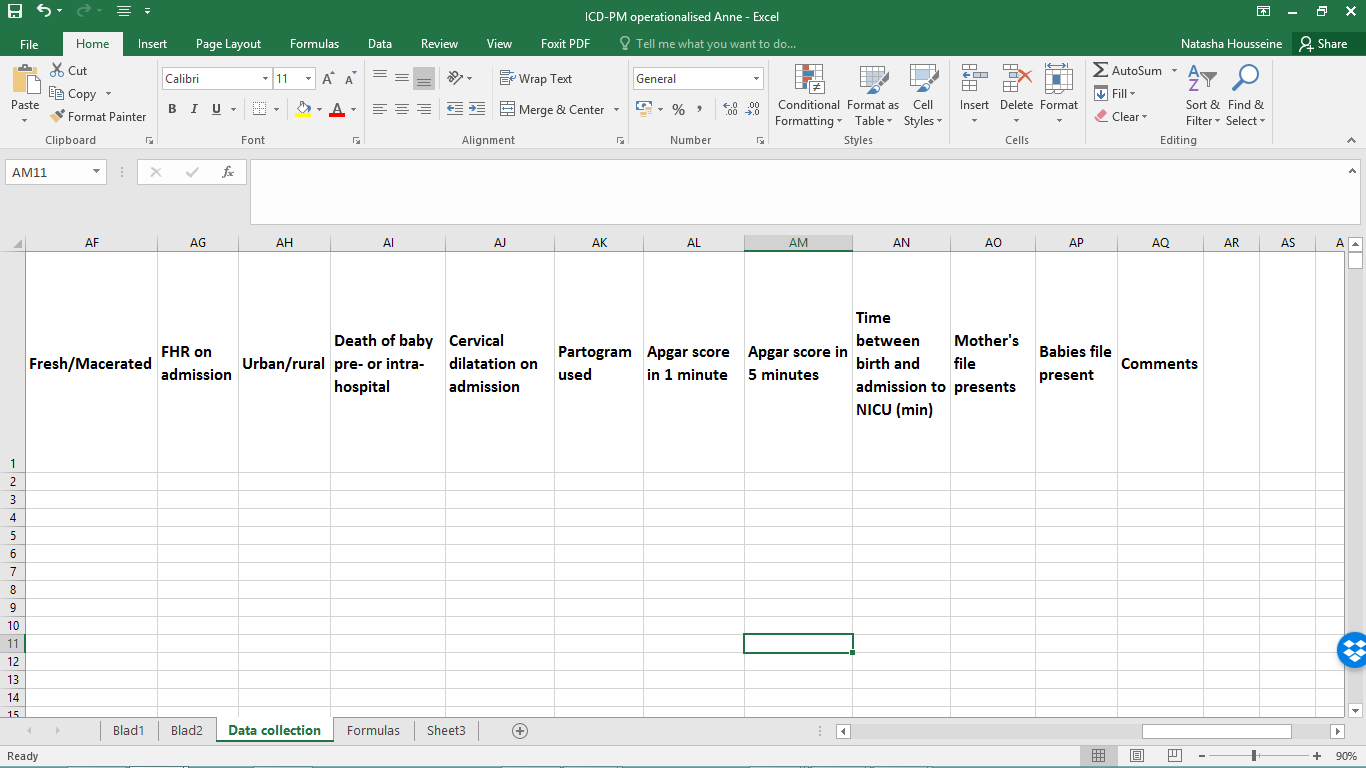

Supplement: S1 Fig — (DOCX) [file pone.0245196.s003.docx]
