# Supplementary material for: The application of WHO ICD-PM: Feasibility for the classification of timing and causes of perinatal deaths in a busy birth centre in a low-income country
Source: PLoS One. 2021 Jan 14;16(1):e0245196. doi: 10.1371/journal.pone.0245196 (PMC7808596; doi:10.1371/journal.pone.0245196)
Supplement: S1 Text — (DOCX) [file pone.0245196.s004.docx]

S1 Text: Examples of cases that illustrate challenges in applying ICD-PM classification

**Unable to classify timing of death**

*Conflicting data of FHR and maceration stillborn baby (fresh/macerated).*

Case 1: A gravida 5 para 4 was admitted in labour pain. On admission, her blood pressure was 135/82, pulse rate 7, haemoglobin of 8.1 g/dL, fundal height was 29cm, FHR of 136bpm and cervix was 3cm dilated. She delivered a macerated baby weighing 1800g. The timing of the death couldn’t be classified for this case, and thus neither the cause of death. No major maternal condition seemed present so the maternal condition assigned was M5: no maternal condition.

*Inadequate intrapartum assessment of women who arrive in early or advanced stage of labour*

Case 2: A prime gravida was admitted to the labour ward with blood pressure was 142/92, pulse 104 bpm and haemoglobin of 13.6. Fundal height was 32cm, FHR was 150bpm and the cervix was closed on admission. No further assessments were documented and it was unknown when labour started and how long after admission she delivered. She delivered macerated stillborn weighing 1700g. The timing of the death couldn’t be classified for this case, and thus neither the cause of death. The maternal complication was classified as M4: maternal medical and surgical conditions (fetus and newborn affected by maternal hypertensive disorders).

Case 3: A gravida 2 para 1 was admitted to the labour ward. She was fully dilated on admission and assessments such as fetal heart rate, blood pressure, haemoglobin measurements were not done. She delivered a stillborn female baby with a birth weight of 2400 grams. The timing of the death couldn’t be classified for this case, neither the cause of death. No major maternal condition seemed present so the maternal condition assigned was M5:no maternal condition identified.

*Only one FHR usually recorded in multiple gestation*

Case 4: A gravida 4 para 3 was admitted in labour pain with a twin pregnancy and gestational age of 40+0 weeks by last menstrual period. On admission, blood pressure was 176/125, FHR was 145 bpm and only recorded for one baby (unknown which one) and cervix was 2cm dilated with a breech presentation of the first twin. The next and last FHR of only one of the babies was recorded 5 hours later when she was 4 cm dilated. The mother delivered vaginally 9 hours after onset of labour. The first baby was alive with Apgar score of 7 at one minute and 9 at ten minutes. The second baby was a macerated stillbirth. Both weighed 2900 g. We were unable to determine timing of the stillbirth as antepartum or intrapartum and also the perinatal cause of death. The maternal complication was classified as M5: maternal medical and surgical conditions (fetus and newborn affected by maternal hypertensive disorders).

**Antepartum deaths of Unspecified cause of death**

Case 5: A gravida 1, para 0 with a gestational age of 30 weeks by fundal height was admitted. On admission, the FHR was absent while the cervix was 1 cm dilated. She delivered a male baby of 2200 g, no information about the appearance of the baby was written down. The timing was assigned as antepartum and the cause of death as A6: foetal death of unspecified cause. No maternal condition was reported so the maternal condition was classified as M5: no maternal condition identified.

**Intrapartum deaths of Unspecified cause of death**

Case 6: A gravida 1 para 0 was admitted to the labour ward. The foetal heart rate on admission was 140 beats per minute and the cervical dilation was 2cm. On the partograph the last foetal heart rate of 130 bpm was recorded 2 hours before delivery when the cervical dilatation was 9cm. A stillborn male baby with a birth weight of 3400 grams was born. It remains unclear what happened between the last recorded foetal heart rate and delivery. The timing for this case was classified as ‘intrapartum’ and the cause of death was assigned as I7: foetal death of unspecified cause since not enough information was available to determine the cause of death. The mother was considered healthy so the maternal condition was classified as M5: no maternal condition identified.

**Variable interpretation of causes of death**

Case 7: A gravida 1 para 0, 40+5 weeks known by using the last menstrual period was admitted to the hospital. During labour there were non-reassuring FHR and thick meconium. A caesarean section was done due to foetal distress. A 2800gram female baby was born with an Apgar score of four in one minute and six in five minutes. The neonate was referred to the NICU where the following vital signs were measured: temperature 34.5 ℃, saturation 73% and a pulse rate 121 beats per minute. Also, meconium secretions were removed from the mouth. The baby started convulsing and de-saturating the same day, she died one day later. The timing of death was classified as early neonatal. There were two possible perinatal causes of death: N4: Complications of intrapartum events (birth asphyxia) and N7: Respiratory and cardiovascular disorders (meconium aspiration). The cause of death was assigned as N4: complications of intrapartum events (birth asphyxia). The mother was considered healthy so the maternal condition was classified as M5: no maternal condition identified.

Case 8: A gravida 3 para 1 presented to the labour ward with a twin pregnancy at 33 weeks of gestation (by LMP). On admission, blood pressure was 134/116 with proteinuria, FHR were absent and cervix was 6cm dilated. She delivered vaginally soon after and both twins were male fresh stillbirths and weighed 1300g and 1500g. Post-delivery the mother was diagnosed with pulmonary oedema due to peripartum cardiomyopathy. The timing of the death was intrapartum. The ICD-PM group of perinatal death was acute intrapartum event (intrauterine hypoxia). There were multiple maternal conditions identified: M2: Maternal complications of pregnancy (twin pregnancy); M3: Other complications of labour and delivery (preterm labour and delivery); and M:4 maternal medical and surgical conditions (Fetus and newborn affected by maternal hypertensive disorders). We chose the latter as the main maternal condition affecting the foetus.

Case 9: A gravida 2 para 1 presented to the labour ward with pregnancy at 37weeks of gestation (by ultrasound). On admission, blood pressure was 173/115 with proteinuria and several symptoms of severe preeclampsia. FHR was 135 bpm and cervix was 3cm dilated. She developed PV bleeding 7 hours after admission. After another 5 hours, she delivered vaginally, male fresh stillbirths and weighing 2500g. The timing of the death was intrapartum. The ICD-PM group of perinatal death was acute intrapartum event (intrauterine hypoxia). There two maternal conditions identified were: severe preeclampsia and abruptio placenta. The maternal complication was classified as maternal medical and surgical conditions, specific group “Fetus and newborn affected by maternal hypertensive disorders”.

**Inability to classify antepartum uterine rupture**

Case 10: A 29-year-old woman, gravida 3 para 2, with a gestational age of 36+4 weeks by ultrasound and 2 previous caesarean sections due to cephalopelvic disproportion presented at the maternity ward with abdominal pain. Foetal heart rate (FHR) on admission was 120bpm and cervix was "tip of finger" dilated. One hour later after admission, patient was examined, vital signs were still stable (BP: 109/64 mmHg, PR:71bpm, SPO2: 98% in room air) but FHR was not detectable, and cervix was still "fingertip" i.e. closed. A decision was made for emergency caesarean section. Intraoperative findings showed the uterus was ruptured at the incision site and the baby's shoulder was out of the uterus. A female baby was extracted, Apgar score of 0 at 1 min and weighed 3000g. We determined timing of death as antepartum and ICD-PM Group for Maternal condition: M3_Other_complications_of_labour_and_delivery;

ICD-PM Maternal condition specific group: P03.8_Fetus_and_newborn_affected_by_other_specified_complications_of_labour_and_delivery_

We understand the classification of this stillbirth under M3 is not ideal. However, uterine rupture does not fit in of any of the ICD-PM Group for Maternal condition for the antepartum period. In this study there were 11 other cases of uterine rupture which occurred intrapartum, we made the decision to categorise all uterine rupture as M3: Other complications of labour and delivery.
